# Large Fibroepithelial Stromal Polyp of the Breast Nipple

**DOI:** 10.7759/cureus.26397

**Published:** 2022-06-28

**Authors:** Sheri P Walls, Olawole Akinboboye, Isaac Opoku, Rafael C Da Silva

**Affiliations:** 1 Internal Medicine, Piedmont Athens Regional Medical Center, Athens, USA; 2 Internal Medicine, Windsor University School of Medicine, Cayon, KNA; 3 Medicine, Universidade Federal de Santa Catarina, Florianopolis, BRA; 4 Medicine, University Center for the Developement of the Alto Vale do Itajaí (UNIDAVI), Rio do Sul, BRA

**Keywords:** polyp, benign tumor, breast cancer, acrochordon, fibroepithelial polyps

## Abstract

Fibroepithelial stromal polyps (FEPs) are benign tumors of the integumentary system with mesodermal origin. They are commonly found over the skin. Rarely, they can be found in the nipple. We report a case in a 62-year-old female with a large FEP emerging from the left breast nipple. It started as a “mole” 10 years ago and slowly progressed in size. The patient did not complain of pain but did report occasional bloody discharge. She has no family history of breast cancer. On physical exam, a pedunculated large polyp, with rubbery consistency, emerging from the left nipple was noted. The lesion consisted of hyper-pigmentation with scattered ulcerations and cauliflower-like growth. Surgical excision was performed and histopathologic analysis showed extensive fibrovascular tissue with dense collagen fibers deposition consistent with FEP. Surgical treatment is curative with minimal skin deformity. In order to make the diagnosis and exclude malignancy, histopathology is mandatory.

## Introduction

Fibroepithelial stromal polyps (FEPs) or acrochordons are mesoderm-derived benign lesions. They are commonly found in the skin, oral cavity, urinary tract, and urogenital tracts. Rarely (incidence <2%), they can be located in the nipple area [1]. FEPs have overlapping morphologic features with mesenchymal lesions of the vulvovaginal area such as leiomyomas, superficial angiomyxoma, cellular angiofibroma, and neurofibromas. Immunohistochemical studies have been described to assist in differentiating these lesions but their clinical value is limited. FEPs rarely grow more than 5 cm; however, lesions larger than 5 cm have been reported [1]. Some experts suggest that FEPs can be estrogen-sensitive placing women at higher risk [2,3]. The aim of this report is to illustrate a rare case of FEPs emerging from an atypical location, the left breast nipple.

## Case presentation

**Figure 1:**
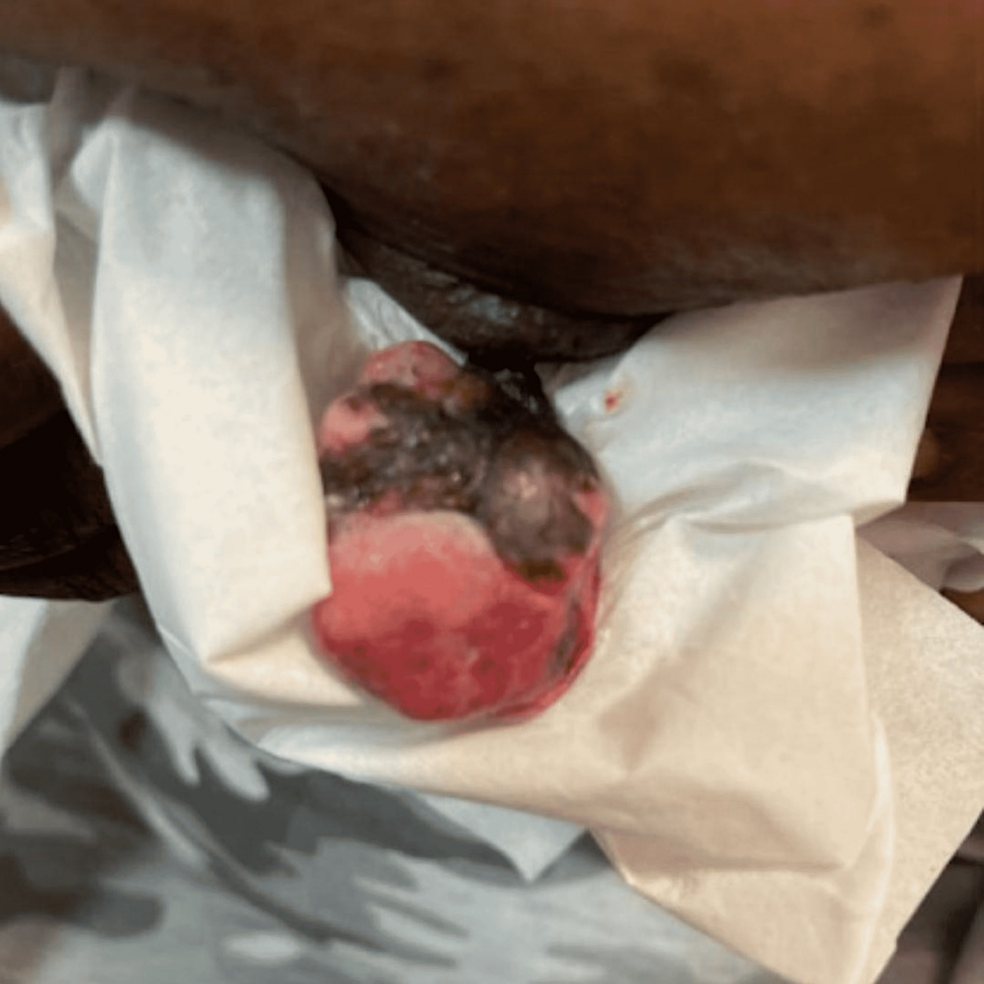
Large pendulous polyp lesion with fibroelastic consistency, short pedunculated emerging from the left nipple. Note areas of superficial erosion.

**Figure 2:**
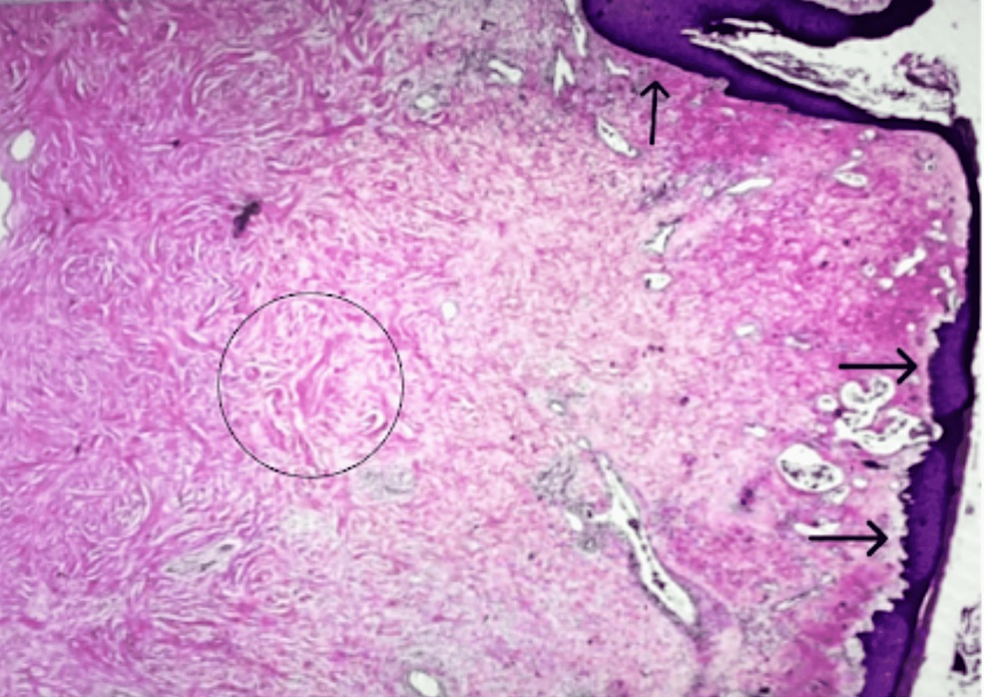
Histopathologic H&E stain 100x showing extensive fibrovascular tissue with dense collagen fibers (black circle) surrounded by a hyperplastic epidermis (black arrows).

A 62-year-old female with a past medical history of hypertension presented with a growing pedunculated exophytic breast lesion, emerging from her left nipple. She stated the breast lesion started as a “mole” 10 years ago, and progressively grew in size. She described it as a nontender, nonpruritic mass, with occasional bloody discharge. She was not on hormone replacement therapy. She has no family history of breast cancer. On review of systems, she denies weight loss, fever, or chills. No other positive or negative pertinent symptoms. On physical examination, a large 3.1 x 2.6 x 2.0 cm short pedunculated polyp lesion, with rubbery consistency, emerging from the left nipple was noted. The polyp had areas of hyperpigmented fleshy-like appearance intercalated with small areas of ulceration and a cauliflower-like appearance (Figure 1). There was no apical or axillary lymphadenopathy. The contralateral breast was unremarkable. A mammogram showed benign findings, Breast Imaging Reporting and Data System I. Patient underwent surgical excision of the lesion and the pathology was consistent with FEP (Figure 2).

 

## Discussion

This report presents a 62-year-old female with a slow-growing large left breast lesion emerging from the nipple, which was ultimately diagnosed as FEP after surgical excision and histopathologic examination. FEPs, also known as acrochordon, are common benign dermal mesenchymal tumors. They are characterized by a polypoid proliferation of the stroma with overlying squamous epithelium that gradually enlarges in size [1,4,5]. Typical histologic features of FEPs include hyperplastic epidermis surrounding a core of fibrovascular tissue with dense collagen fibers [4,6]. They are commonly seen in women of reproductive age, often during pregnancy or in postmenopausal women under hormone replacement therapy. They are primarily seen in the vagina but can also occur in the vulva or cervix [2,7]. Our patient presents with FEP in a rare location, with atypical features such as ulceration and intermittent bleeding. One hypothesis for the areas of ulceration and intermittent bleeding is the association with microtraumas caused by continuous attrition of the lesion and the clothes used by the patient [7]. Through a review of the literature using the terms “breast” AND “nipple” AND “fibroepithelial” AND “polyp” only a few reports could be retrieved. Despite the majority of the FEPs being considered benign tumors, they still have a small potential for malignancy [6]. In a previous study examining 1,335 clinical specimens, there were five reported malignant tumors, whose relative risk was 0.4 [6]. However, this was a study examining all fibroepithelial polyps and not specifically FEPs of the nipple. The main concern for patients presenting with FEPs is that other common malignancies such as intraductal papilloma, leiomyoma, and metaplastic (spindle cell) carcinoma may have similar presentations, so it is important to undergo histopathological analysis for definitive diagnosis [4,7,8].

## Conclusions

Fibroepithelial polyps are common skin benign tumors and rarely occur in the female nipple. Clinically, those types of benign tumors, as in this case can present as a slow-growing pedunculated tumor, with a rubbery, nodular, or cauliflower-like appearance. Occasionally, they can ulcerate and cause bloody discharge. Histologically, FEPs are characterized by hyperplastic epidermis surrounding a core of fibrovascular tissue with dense collagen fibers. Intraductal papilloma and squamous cell carcinoma are differential diagnoses. Surgical resection is curative.
